# Bilateral Anterior Lingual Depression in the Mandible: Cone Beam Computed Tomography Case Report and Review of the Literature

**DOI:** 10.7759/cureus.6348

**Published:** 2019-12-11

**Authors:** Manea Altwaim, Ra’ed Al-Sadhan

**Affiliations:** 1 Dentistry, King Saud University, Riyadh, SAU; 2 Oral and Maxillofacial Surgery, College of Dentistry, King Saud University, Riyadh, SAU

**Keywords:** mandible, lingual, sublingual, fossa, depression, implant, complication

## Abstract

The mandibular anterior lingual depression is an uncommon anatomical variant. It is difficult to be detected in conventional 2D plain radiographs representing a diagnostic challenge. In this report, we describe a patient who presented to the dental clinic for the extraction of impacted third molars. Upon cone beam computed tomography (CBCT) examination of the mandible, it was incidentally noted that he had bilateral anterior lingual depressions in his mandible. The presence of anterior lingual depressions is uncommon and to be found bilateral is rare. This bone topography represents a challenge for the oral surgeon during implant placement with an increased risk of complications. The incidental finding was documented in the patient's dental record for future implications in case an implant placement was needed.

## Introduction

The restoration of missing teeth to resume proper function is important for maintaining the quality of life [1]. The treatment modalities available for tooth replacement has changed over the years with dental implants becoming the standard of care supported by raising evidence of long term success [1]. Comprehensive evaluation and treatment planning ensures predictable and satisfactory outcome [2]. Implant site assessment with conventional plane 2D images such as periapical and panoramic radiographs has limitations related to distortion and anatomical superimposition [3-4]. The American Academy of Oral and Maxillofacial Radiology recommends the use of CBCT for implant site assessment and treatment planning, this recommendation is emphasized in the presence of limited bone quantity as evident by clinical evaluation [5]. In the mandible, the interforaminal area located between the right and left mental foramen is used frequently for implant placement. Difficulties may arise if the anatomical boundaries are violated and anatomical structures are jeopardized. Immediate implant placement complications include bleeding, nerve damage, injury to adjacent teeth, and implant displacement into neighboring anatomical spaces [6-7]. If postoperative bleeding occurred in the interforaminal area of the mandible, it could spread to the adjacent parapharyngeal and retropharyngeal spaces, leading to life-threatening complications [7-10]. The risk of hemorrhage is highest in the anterior mandible due to the high vascularity of the floor of the mouth and the bone topography such as concavities or fossa in this area [7]. The presence of lingual concavities in the anterior mandible carries a potential higher risk of perforation and the complication sequelae [11-12]. The Lingual bone concavities are hypothesized to arise due to the pressure exerted by the adjacent sublingual and submandibular glands [13]. Following extraction, alveolar bone resorption occurs in a predictable pattern leading to the sloping of the crestal bone to the lingual side, which increases the risk of perforation when using bigger diameter drills and implant [1,5,14]. Parnia et al. proposed a classification of the submandibular concavities based on morphology and found that the increased depth was associated with a higher risk of perforation [15]. No such classification can be found for sublingual depressions in the lingual surface of the anterior portion of the mandible beyond the submandibular concavities.

This report aims to describe a case exhibiting bilateral anterior lingual concavity in CBCT. The review of the literature highlights its significance in implant treatment planning and prediction of surgical complications.

## Case presentation

**Figure 1:**
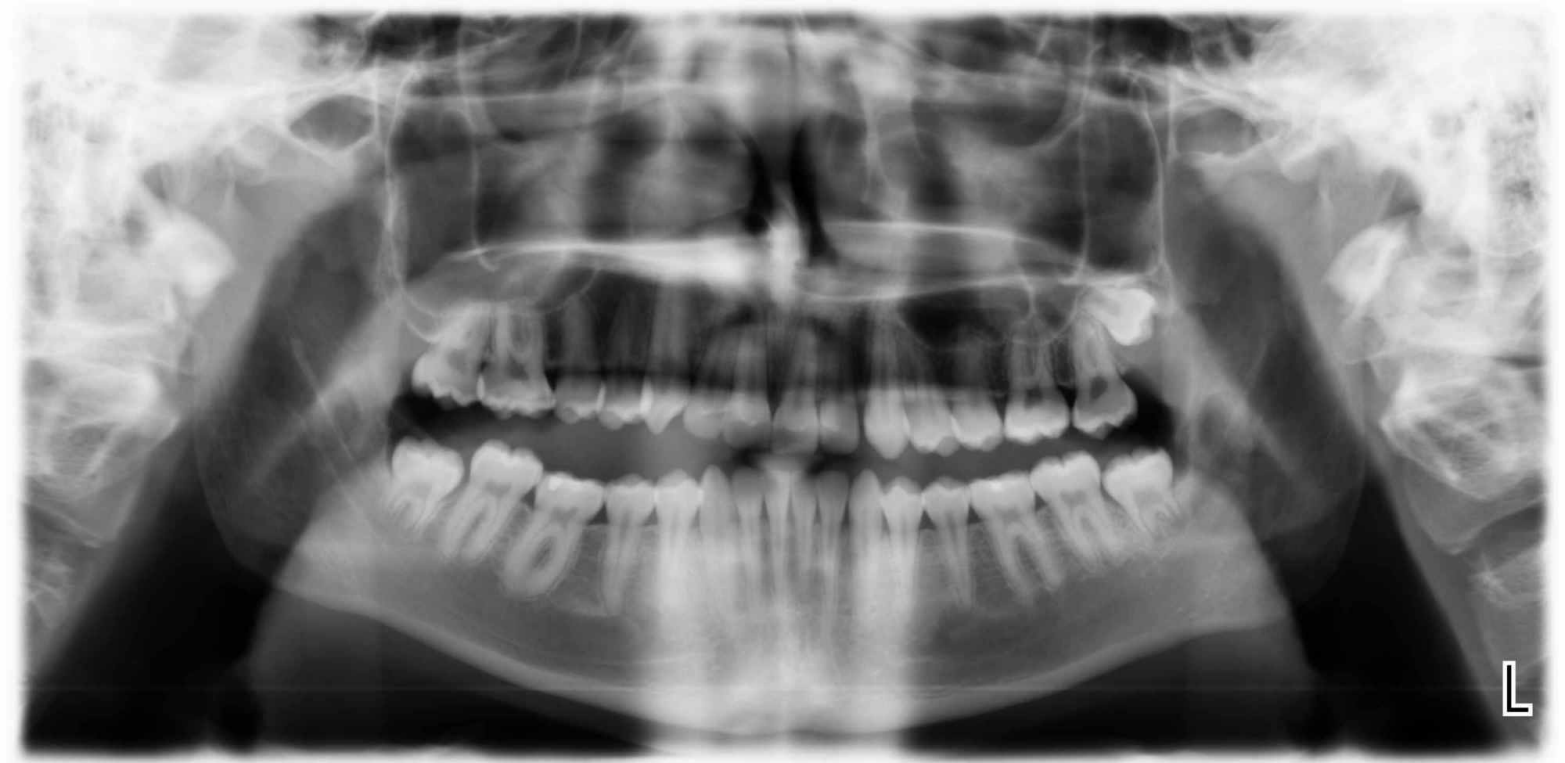
Panoramic radiograph

A 17-year-old male patient attended the oral and maxillofacial surgery clinic in King Saud University requesting extraction of all third molars. The patient’s medical history was unremarkable; he was a non-smoker and revealed no history of previous facial trauma or surgery. There was neither pain nor swelling of the buccal and lingual sulci of the mandible and no cervical lymphadenopathy. Initial dental examination revealed a minimally restored permanent dentition with good oral hygiene. A panoramic radiograph showed that all third molars were present and impacted except on the upper right side where the tooth was missing, with the lower third molars in close proximity to the inferior alveolar canals (Figure 1). The radiograph was otherwise unremarkable with no signs of pathology or abnormality.

A CBCT of the mandible was requested for further assessment of the relation and proximity of the roots to the inferior alveolar canal. A survey of the CBCT sections of the mandibular body revealed bilateral sublingual depressions inferior to the premolars and first molars extending to the inferior border of the mandible that was not seen on the panoramic radiograph.

**Figure 2:**
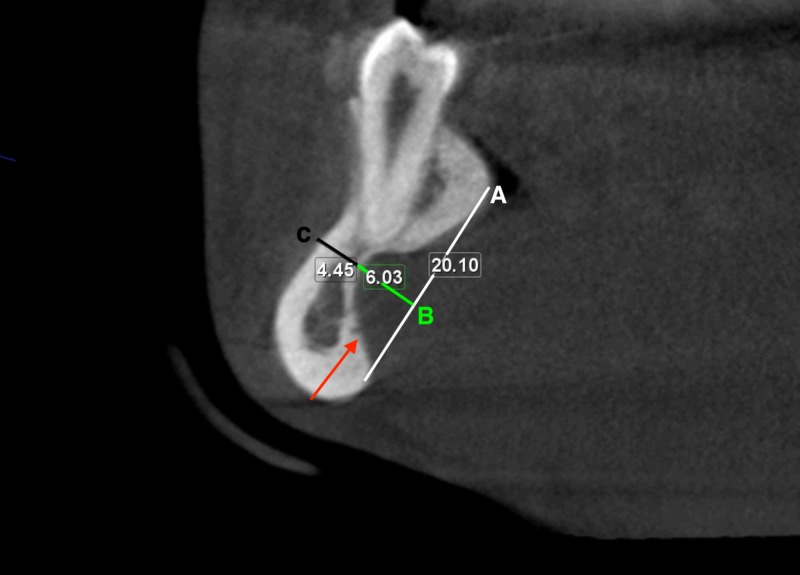
Cross-sectional cone beam computed tomography of the right lingual depression Lingual depression (red arrow), line tangent to the heights of contours of the lingual surfaces of the mandible (A), the deepest point of the depression (B), the buccolingual thickness of the remaining mandible (C).

**Figure 3:**
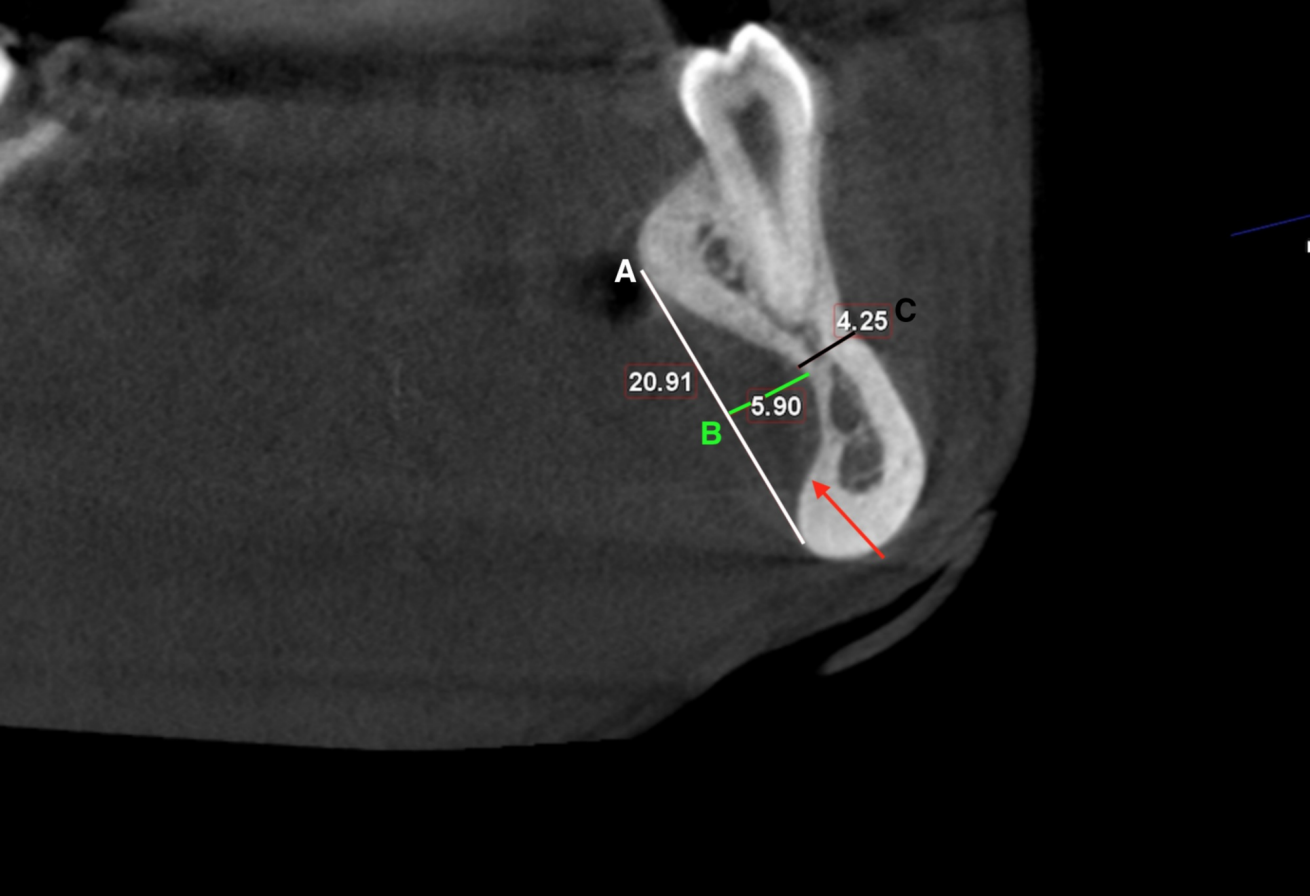
Cross-sectional cone beam computed tomography of the left lingual depression Lingual depression (red arrow), a line tangent to the heights of contours of the lingual surfaces of the mandible (A), the deepest point of the depression (B), the buccolingual thickness of the remaining mandible (C).

To assess the depth and width of these depressions, the CBCT images were reconstructed in planes aligned with the axes of the right and left mandibular second premolars in the mesiodistal dimension, buccolingual dimension, and the coronoapical dimension. Then, linear measurements representing the width of the depressions were made along a line drawn a tangent to the heights of contours (crest of curvatures) of the lingual surfaces of the mandible superior and inferior to the bilateral depression. The superoinferior width of the depressions was found to be 2.1 cm on the right side and 2.9 cm on the left side. Linear measurements representing the buccolingual depth of the depressions were made along a line drawn perpendicular to the above-mentioned width line to the deepest point of the depression; it measured 0.59 cm on the left side and 0.6 cm on the right side. The buccolingual thickness of the remaining mandible was 0.42 cm on the left side and 0.45 cm on the right side (Figures 2-3).

The diagnosis was concluded to be bilateral sublingual depressions of the mandible. It is considered a normal anatomical variation that does not require any further investigation or intervention. The incidental finding was documented in the patient dental record for future implications in case an implant placement was needed.

## Discussion

The patient in the aforementioned case report did not have any history of trauma or surgery in the region. The radiographic evidence of lingual depression was found incidentally and the indication for CBCT request was irrelevant to the findings with no clinical signs or symptoms. An anomaly with a similar presentation in the mandible is the Stefan bone cyst, first reported by Edward Stafne in 1942 [16]. The report mentioned 35 cases of unilocular radiolucent lesion appearing in the posterior mandible [16]. In 1957, Richard and Ziskind reported on a lingual boney concavity located in the mandibular canine and premolar region that they have explored surgically [4]. Since then more topographic variants have been described and mandibular depressions are generally categorized into posterior mandible lingually, anterior mandible lingually, mandibular ramus lingually and mandibular ramus buccally [13]. Harvey and Noble presented a cadaveric and histologic examination of mandibular lingual cortical defects and found that the depth of the concavity in relation to the surrounding bone determined its appearance on plane radiographs and hence clinical appreciation as Stefan’s cyst [17]. Previous reports of excised lesions showed evidence of salivary gland tissue, adipose tissue, blood vessels, smooth muscle, connective fibrous tissue or lymphoid tissue [18-19].

A common differential diagnosis of the lesion, when first discovered on plane (panoramic) radiographs, is a periapical cyst because of the proximity of the defect to the roots of incisor and premolar teeth [13]. The defect is considered an anatomical anomaly rather than a true pathology requiring conservative management with serial imaging follow up rather than surgery [13]. The clinical significance of the lesion is recognized during implant treatment planning, as the presence of the depression is associated with the possible risk of near-fatal complications. Interforaminal implant placement is generally considered a safe procedure with low risk for complications. However, intraoperative bleeding in the floor of the mouth can result in sublingual hematoma formation raising the tongue and obscuring the airway as seen in the Pseudo-Ludwig phenomenon which is an airway emergency that is a constant threat to life [7-12,20]. Kalpidis reported 12 cases of implant surgical complications resulting in an impingement of the airway and advised precautionary measures relate to a comprehensive evaluation of the site anatomy, implant position and angulation, and cautious surgical site preparation [2]. Other reported complications with later onset following implant perforation of the lingual plate include infection that can spread beyond the mandibular region leading to mediastinitis, mycotic aneurysm, and ultimately airway embarrassment [10].

## Conclusions

The anterior lingual depression is a rare anatomic variant that represents a challenge during implant practice. Due to its location, the depression can be easily missed on panoramic or periapical radiographs but can readily be identified by the CBCT examination, thereby emphasizing the importance of this imaging modality for implant site assessment and treatment planning in implant dentistry to prevent serious and life-threatening complications.
